# Perspectives on Successful Aging Across Diverse Cultural and Social Contexts

**DOI:** 10.1093/geroni/igaf122.698

**Published:** 2025-12-31

**Authors:** Emily Willroth, Payton Rule

## Abstract

Promoting successful aging is a global priority (e.g., World Health Organization, 2022). However, perceptions of successful aging can differ significantly across cultural and social contexts. Understanding these diverse perspectives is crucial for promoting successful aging for all individuals. This symposium features research on perspectives of successful aging from underrepresented groups, including LGBTQ individuals, people in low- and middle-income countries, individuals with disabilities, and those from diverse racial, ethnic, and cultural backgrounds. First, Dr. Fredriksen Goldsen will present results from a longitudinal study identifying life affirming pathways to sexual and gender diverse older adults’ wellbeing and good health. Dr. Ivan will discuss the impact of e-health literacy on reducing loneliness and sustaining wellbeing during the pandemic, comparing middle- and high-income countries. Rule will present findings from a study examining older adult perceptions of a new, more disability-inclusive definition of successful aging. Lewis will discuss healthy aging experiences among Black women. Lastly, Dr. Nguyen will present findings from a study investigating how individual notions of successful aging can be linked to, and co-constituted by, relational and intergenerational notions of personhood within the broader socio-economic, familial, and cultural contexts of migration. In sum, this symposium presents evidence for differences in definitions of and pathways to successful aging across cultural and social contexts.

